# Clot Composition and Ischemic Stroke Etiology: A Contemporary Narrative Review

**DOI:** 10.3390/jcm14176203

**Published:** 2025-09-02

**Authors:** Jacob Kosyakovsky, Christina P. Rossitto, Joseph P. Antonios, Daniela Renedo, Christopher J. Stapleton, Lauren H. Sansing, Dhasakumar S. Navaratnam, James A. Giles, Aman B. Patel, Charles C. Matouk, Nanthiya Sujijantarat

**Affiliations:** 1 Department of Neurosurgery, Mass General Brigham, Harvard Medical School, Boston, MA 02115, USA; jkosyakovsky@mgb.org (J.K.); crossitto@mgh.harvard.edu (C.P.R.); cstapleton@mgh.harvard.edu (C.J.S.); abpatel@mgh.harvard.edu (A.B.P.); 2 Department of Neurosurgery, Yale-New Haven Hospital, Yale University, New Haven, CT 06510, USA; joseph.antonios@yale.edu (J.P.A.); daniela.renedo@yale.edu (D.R.); 3 Department of Neurology, Yale-New Haven Hospital, Yale University, New Haven, CT 06510, USA; lauren.sansing@yale.edu (L.H.S.); dhasakumar.navaratnam@yale.edu (D.S.N.); james.giles@yale.edu (J.A.G.); 4 Department of Radiology, Yale-New Haven Hospital, Yale University, New Haven, CT 06510, USA

**Keywords:** ischemic stroke, clot composition analysis, mechanical thrombectomy, cryptogenic stroke, multiomic sequencing

## Abstract

Acute ischemic stroke (AIS) is one of the leading global causes of mortality and morbidity. Clearer understanding of stroke etiology is a major clinical objective to determine appropriate strategies for secondary stroke prevention. Histological and molecular analysis of clots retrieved during mechanical thrombectomy (MT) in AIS offers a unique opportunity to study clot composition and its relation to stroke etiology. The field of clot composition analysis has undergone substantial growth in recent years, driven in part by the establishment of MT as the standard of care, as well as its expanding indications. Although many features differ between large-artery atherosclerosis (LAA) and cardioembolic (CE) clots, application of these findings to predicting stroke etiology at a clinical level remains challenging. Moreover, a significant number of patients have multiple comorbidities or suffer a cryptogenic subtype. Next-generation techniques such as multiomic sequencing offer a powerful potential to elevate our understanding of clot pathology and provide the level of granularity required for clinical diagnosis and management. Herein, we provide an updated review of the current state of the field by exploring stroke etiologies and their relationship to clot pathology, including classic histologic features as well as more recent, emerging results from proteomic and transcriptomic analyses.

## 1. Introduction

Acute ischemic stroke (AIS) is one of the leading causes of global mortality and morbidity [1,2]. Fibrinolysis with tissue plasminogen activator (tPA) or, most recently, tenecteplase (TNK) remains the first-line treatment for AIS patients meeting eligibility criteria [3,4]. The eligibility criteria include, but are not limited to, adult patients with presentation up to 3 or 4.5 h of onset, and absence of contraindications such as intracranial hemorrhage, recent surgery, or known coagulopathy [5]. Large-vessel occlusion (LVO) represents a subset of AIS cases whereby blockage occurs in a proximal vessel, and is estimated to be responsible for 7.2% of stroke code alerts [6], and 4.9% in patients with suspected stroke symptoms, by emergency medical technicians (EMTs) [7]. Over the past decade, the management of LVO has been transformed by widespread adoption of endovascular therapy with mechanical thrombectomy (MT) [8]. Beginning in 2015, a series of landmark trials established MT as the standard of care for patients with LVO presenting up to 8 h of last known well (LKW) [9,10,11,12,13]. Subsequent trials expanded the treatment window up to 24 h [14,15,16]. Today, patients meeting inclusion criteria are simultaneously triaged for both medical and endovascular intervention.

Beyond acute care, effective stroke management depends on accurate identification of etiology to guide secondary stroke prevention. The TOAST classification [17] provides a clinical framework to categorize stroke etiology and is detailed in Table 1. While cardioembolism (CE) represents a major identified cause [18], it is not uncommon for patients to have multiple comorbidities, as the risk factors for these conditions frequently overlap [18]. Additionally, cryptogenic strokes were estimated to be responsible for up to 30–40% of patients [19]. Because the risk of recurrent stroke is high, up to 30% within ten years [19], these scenarios can pose a major clinical challenge in secondary stroke prevention. The search for clinical or biological indicators of stroke etiology has been the focus of substantial research effort, given its direct implications for clinical management [20].

MT, especially with the popularization of direct aspiration techniques, has provided a unique opportunity to study clot pathology [21]. The retrieval of clots that would otherwise be inaccessible enables “endovascular biopsy” [22], which offers insights into the mechanisms responsible for these LVOs. This review aims to examine the most current literature exploring the relationship between clot composition and stroke etiology to date. We summarize key studies comparing large-artery atherosclerotic (LAA) and CE clots using traditional hematoxylin and eosin (H&E) staining, with particular attention to the ongoing debate surrounding “red” vs. “white” clots. We then discuss white blood cell (WBC) content and its substructure, including neutrophil extracellular traps (NETs). Finally, we highlight emerging molecular approaches, including proteomic, metabolomic, and transcriptomic analyses, which offer new insights into clot characterization and represent the next frontier in this field.

## 2. A Primer on Stroke Etiology and Clot Pathology

**Figure 1:**
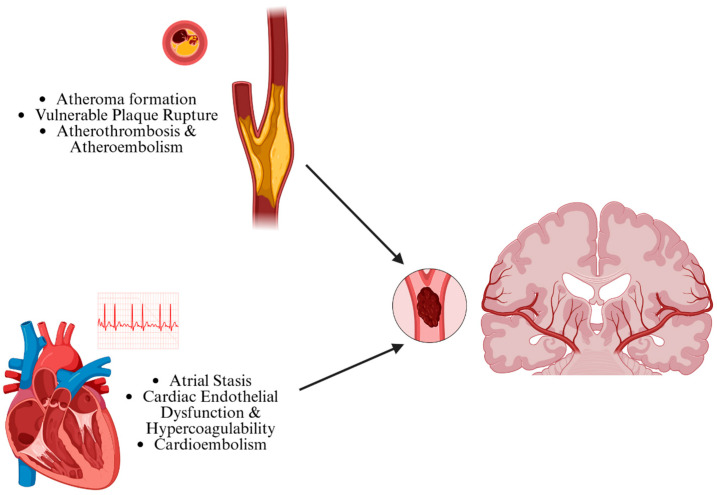
A simplified schematic showing pathophysiological processes leading to atheroembolism and cardioembolism, the two primary causes of large-vessel occlusion (LVO). Created in BioRender. Sujijantarat, N. (2025) https://BioRender.com/tb9f0wn (accessed on 9 August 2025).

Each stroke subtype reflects a distinct underlying disease process and necessitates a tailored approach for secondary stroke prevention. Because retrieval of clots is only possible for TOAST subtypes resulting in LVO, lacuna strokes and small-vessel disease (SVD) cannot be studied, except in the post-mortem period. Prior studies of SVD have implicated multifocal mechanisms of stroke involving endothelial dysfunction, blood–brain barrier breakdown, and lacunar infarction [17,23,24]. Postmortem analyses have further demonstrated microglial and endothelial activation in affected tissue [25,26,27]. In the context of LVO, the most-encountered etiologies are LAA or CE, which reflect distinct disease processes (Figure 1). As will be discussed in the following section, growing evidence suggests that cryptogenic strokes may share similar mechanisms with CE clots.

Atherosclerosis is characterized by the buildup of fatty atheromatous plaques within blood vessel walls and has a number of well-established epidemiological risk factors, including smoking, hypertension, hyperlipidemia, obesity, and diabetes [28]. In response to a variety of pro-inflammatory signals, the intima, the innermost arterial layer, begins to recruit inflammatory cells, including lipid-laden macrophages or foam cells [29]. Over time, this intimal lesion containing inflammatory cells, smooth muscle, and extracellular matrix may expand into an enlarging atheroma [29]. This accumulation gives rise to a classic lipid-rich, necrotic core [30]. Rupture of the inflammatory caps of vulnerable atheromatous plaques can then expose the inner pro-thrombotic, inflammatory plaque microenvironment to circulating platelets and fibrin, which incites clot formation [29]. In combination with vessel aging [31], subsequent stenosis and impaired vascular dynamics [32], plaque rupture, and thrombus formation can embolize downstream, resulting in LAA LVO.

In contrast to LAA LVO, LVO associated with CE is a result of thromboembolism of a clot generated in the heart. Atrial fibrillation represents the most common etiology of CE and involves impaired coordination of atrial contraction, leading to stasis of blood flow. This stasis, one of the components of Virchow’s triad, predisposes to clot formation, which can then embolize to the intracranial circulation [33]. Hypercoagulability and endothelial dysfunction—the remaining components of Virchow’s triad—may also interact to promote inflammation [34]. While CE shares similarities to LAA, the cardiac environment in which thrombus formation occurs is associated with important differences in biology [35]. Other causes of CE include the presence of a prosthesis such as a mechanical heart valve, or iatrogenic factors such as during cardiac procedures.

Clots retrieved during MT exhibit cellular and molecular profiles that reflect their underlying pathology [36]. On traditional hematoxylin and eosin (H&E) staining, microscopic analysis of extracted clots allows for identification and quantification of cellular clot subcomponents, including red blood cells (RBCs), platelets, white blood cells (WBCs), and coagulation proteins, including fibrin and von Willebrand factor (vWF). Special stains enable detailed WBC subtyping and facilitate the detection of NETs. Transcriptomic signatures within the clots or specific cellular components can be analyzed using RNA sequencing. Protein expressions involved in clot pathogenesis can be quantified through mass spectrometry or other proteomic techniques. Together, these approaches to clot composition provide insight into the underlying pathology and, in turn, uncover clues about the etiologic “past” of each clot [37].

## 3. Insights from Red Blood Cell, Fibrin, and Platelet Analysis

H&E has long served as the standard approach for composition analysis and has the ability to detect RBCs, fibrin, and platelets, among other components [38]. Traditionally, venous clots were thought to be rich in RBCs (“red clots”) and poor in platelets/fibrin, whereas arterial clots were poor in RBCs and rich in platelets/fibrin (“white clots”) [30]. However, recent studies have revealed that arterial clots can also contain a significant portion of RBCs, and that significant variability exists in the histological appearance and composition of LVO clots [38]. These insights prompted growing research interest in clot composition and its ability to differentiate between different stroke etiologies.

A range of staining techniques and quantification methods have been described in the literature [20]. While the majority of studies utilized H&E staining, Martius blue staining is also commonly used as a more specific technique to assess RBCs and fibrin. Elastica van Gieson, Prussian Blue, and other staining techniques have also been described [21]. Various immunohistochemical (IHC) stains can be used to quantify vWF, fibrin, and other components. For example, RBCs can be selectively quantified via CD235a (glycophorin) antigen [21], while platelets can be labeled with CD41, CD42b, or CD61 [21]. Once staining is completed, quantification is achieved either manually or with the aid of software automation [39].

The distinction between “red” and “white” clots in differentiating between LAA and CE etiologies remains a subject of debate. While some studies reported an increased proportion of RBCs compared to platelets/fibrin (“red clot”) in LAA compared to CE clots, others reported the opposite results [40,41,42]. In recent systematic reviews and meta-analyses, RBCs were found to be more prevalent compared to platelets/fibrin among the LAA clots, whereas CE clots tended to have more platelets/fibrin [39,43]. Significant heterogeneity existed among the included studies, likely owing to differences in staining techniques and reporting, as well as the presence of confounders such as the use of intravenous (IV) thrombolysis and specific MT methods [39,43]. In the most recent large-scale quantitative histological study of 501 clots, Staessens et al. found a statistically significant difference in RBC components between LAA and CE clots, with RBCs comprising 52% in LAA clots vs. 38% in CE clots [40].

On the other hand, an increased presence of platelets/fibrin (“white clot”) has been found to be more common when CE is the underlying etiology. In a meta-analysis from our group, CE clots were found to have approximately 10% more platelets/fibrin compared to LAA clots across the 12 studies included [39]. Similarly, Huang et al. found significantly more platelets/fibrin in the CE clots across the studies included [43]. Significant heterogeneity was again noted across studies [39,43]. Although some studies reported platelets and fibrin separately, many listed combined compositions due to limitations in staining and quantification techniques [39,43]. In the Staessens et al. study, CE clots comprised of 34 ± 17% fibrin and 37 ± 18% platelets, whereas LAA clots only contained 25 ± 13% and 29 ± 14% of these components, respectively [40].

Importantly, many studies have highlighted that the clot composition of cryptogenic stroke tended to mirror that of CE clots [20]. In an analysis of 187 patients with LVO, including 64 with cryptogenic strokes, Sporns et al. found that the composition of cryptogenic stroke was similar to that of CE, and that they both tended to have more fibrin and fewer RBCs compared to LAA clots [44]. Similarly, Boeckh-Behrens et al. studied a cohort of 145 consecutive LVO cases including 36 cryptogenic clots [45]. Platelet/fibrin composition was similar between cryptogenic and CE clots and was greater in proportion compared to non-CE clots. RBCs were again found to comprise a smaller portion of cryptogenic and CE clots compared to non-CE [45]. In their meta-analysis, Huang et al. reported that cryptogenic strokes had fewer RBCs, more fibrin, and no significant difference in platelet content compared to LAA strokes [43]. Staessens et al. found similar relationships between cryptogenic, CE, and LAA clots with regards to platelet and RBC content [40]. They identified significantly more platelets and fewer RBCs in cryptogenic and CE compared to LAA clots, but were not able to detect a statistically significant difference in the fibrin content. These histopathological associations have led to a speculation that cryptogenic strokes may be CE in nature, including atrial fibrillation undetected during the hospital admission or outpatient monitoring [19,40,45]. Although hypothesis-generating, these associations may not reliably predict the etiology of individual clots given considerable heterogeneity even within clots of the same etiologic origin [39,40].

Despite observable differences between CE and LAA clots on H&E at a population scale, their clinical relevance remains limited [39]. This clinical limitation is attributable to relatively minor differences between groups (~10% comparing platelet, RBC, or fibrin composition) and significant intra-group variability [39,40,45,46]. For example, in a study analyzing 662 CE clots and 267 LAA clots, Brinkinji et al. found statistically significant differences in RBC, platelet, and fibrin content between the groups. However, they were unable to define a reliable threshold to differentiate the two etiologies based on H&E analysis alone [46]. Likewise, although Staessens et al. were also able to define differences in H&E clot composition between stroke etiologies, they were unable to create a successful multivariate model that explained stroke etiology based on clot composition [40]. As a result, translating H&E-based differences in clot composition into clinical tools for determining stroke etiology on an individual basis remains challenging [39,40].

## 4. Insights from White Blood Cells and Neutrophil Extracellular Trap

Inflammation and immune cell infiltration are believed to play a key role in clot formation both in LAA [29] and CE [34]. Various histologic techniques have been employed to identify inflammatory cells within clots retrieved during MT. For instance, CD45 serves as a marker for total WBC content [21], while subtype-specific immunohistochemistry (IHC) staining enables characterization of individual WBC subpopulations. Neutrophil subpopulations can be identified using markers such as neutrophil elastase (NE), neutrophil myeloperoxidase (MPO), or CD66b [21]. CD3 and CD20 are used to label T cells and B cells, respectively, with CD4 and CD8 for their respective T cell subpopulations [21]. Monocytes can be identified using CD14, while CD68 labels both monocytes and macrophages [21]. In addition to cellular markers, neutrophil extracellular trap (NET)—a fibrous structure composed of extracellular DNA released by activated neutrophils—is commonly observed in the stroke clots [47] and can be quantified using IHC staining for citrullinated histone H3 (H3Cit) [21] or via direct extracellular DNA quantification [40]. Table 2 summarizes common techniques used to detect clot compositions and WBC subpopulations.

Total WBC composition has also been investigated for its possible correlation with stroke etiology [38,40,44,46]. In a meta-analysis from our group, a 1% pooled mean difference was found in the total WBC composition between the CE and non-CE/LAA groups, with the CE group containing a statistically higher proportion of WBCs [39]. Although statistically significant, this number is unlikely to be clinically meaningful. On the other hand, the meta-analysis of eight studies by Huang et al. [43] included two studies with conflicting results on the relative enrichment of WBCs in LAA [69] vs. CE clots [44] and reported no statistically significant difference. The majority of original studies failed to detect significant differences in WBC populations [43]. Similar to the trend seen in RBC and platelet/fibrin components where cryptogenic clot mirrored the CE clots, more WBCs were found in cryptogenic vs. LAA clots, although this difference did not reach statistical significance [43]. The results from the Staessens et al. study were in line with previously published literature [39], with more WBCs in CE compared to LAA clots (20 ± 14% vs. 15 ± 14%, respectively) [40]. Interestingly, overall WBC percentages have not been found to be clearly different in the clots of patients with concomitant active cancer [70] or in atypical clots such as fat or septic emboli [71].

The clinical utility of correlating the quantity of WBCs with specific stroke etiologies is limited. However, differentiation of WBC subpopulations has been examined as a potential tool to distinguish between stroke etiologies. Both Sporns et al. [44] and Goebel et al. [52] found a trend towards higher CD68+ monocytes/macrophages in CE clots, while Dargazanli et al. found that CD3+ lymphocytes were more common in LAA clots [66]. Juega et al. reported higher proportions of natural killer (NK) cells and CD4+ T cells, but not CD8+ T cells, in LAA clots [72]. Similarly, Pagola et al. found an increased prevalence of CD4+ T cells in LAA compared to CE clots [73]. Essig et al. and Novotny et al. reported greater neutrophil concentrations in CE clots [58,64]. Thus, CE clots appear to have a greater involvement of innate immune cells such as monocytes, macrophages, and neutrophils, whereas LAA clots tend to have greater lymphocytic infiltration. These conclusions are supported by a recent study by Jabrah et al., who found that LAA clots had increased lymphocytic infiltration compared to both CE and cryptogenic clots, whereas CE clots had increased CD66b+ neutrophils [74]. Of note, increasing evidence highlights the importance of adhesion molecules (e.g., E-selectin, P-selectin, and L-selectin) in mediating thrombosis and inflammation. For example, E-selectin, expressed exclusively on endothelial cells, was found to be upregulated in ischemic vasculature shortly after reperfusion [75]. P-selectin, on the other hand, mediates the interplay between platelets and leukocytes on activated endothelial cells [76] and shows distinct spatial organization within AIS clots: P-selectin-positive platelets were concentrated in the core, whereas less dense P-selectin-negative platelets were found to be located in the shell [77,78]. Although a full discussion of these adhesion molecules is beyond the scope of this review, the primary literature offers important insights into their crucial roles and is well worth further reading. Table 3 summarizes the current evidence to date on WBC subpopulations in LAA and CE clots.

In the past decade, NETs have been widely implicated as a contributor to thrombosis [20,47,79]. NET formation, known as “NETosis,” involves localization of pro-coagulant factors and histone citrullination, which leads to decondensation of the nuclear chromatin and subsequent release of fibrous extracellular network [80,81,82]. Originally identified as a neutrophil-mediated immunity, this process forms a scaffold that facilitates platelets and RBC aggregation, and has been implicated in the formation of both venous and arterial clots [82]. Platelet activation, in part modulated through P-selectin, accelerates NETosis and contributes to inflammation, thrombosis, and ischemic injury [83]. NET burden was found to be associated with increased inflammation in the left atrium [37,84,85], predisposing to clot formation and subsequent cardioembolism. In a recent study, NET formation represented a key source of extracellular DNA that triggered activation of the AIM2 (Absent in melanoma 2, a dsDNA-triggered) inflammasome after an initial stroke [86]. In turn, increased inflammation in vulnerable plaques leads to arterial embolism and recurrent stroke. Remarkably, elimination of extracellular DNA by DNase treatment has been shown to promote ex vivo clot lysis [47] and reduce the rate of stroke recurrence after experimental stroke [86]. This leads some authors to speculate whether DNase treatment administration with IV thrombolysis could potentially be beneficial in patients with CE LVO [40]. Although there are inconsistencies in the reported neutrophil content between CE and LAA clots [58,64,74], NETs and extracellular DNA have been consistently reported in greater quantity in CE compared to LAA clots across a number of studies [20,40,47,58]. Furthermore, NETs appear to exhibit a more peripheral, organized distribution in CE clots, as opposed to the more diffuse pattern seen in LAA clots [74]. The presence of NETs also serves as an indicator of age [37,47,87], with fresh clot containing less NETs, which increases in content as the clot matures [47,87]. Because fresh clot is typically associated with improved thrombolysis in cerebral infarction scores (TICI) and decreased procedural time [87], it is not surprising that the presence of NETs has been shown to be associated with worse outcome [58]. The increased presence of NETs in CE clots may reflect underlying differences in pathogenesis across stroke etiologies. One possible explanation is that CE clots may have greater exposure to NETosis and NET formation prior to embolization, whereas LAA clots may form more acutely in the setting of plaque rupture, resulting in comparatively less NET formation. These insights remain speculative and require additional validation.

## 5. Molecular and Next-Generation Analysis

The repertoire of techniques for analyzing clinical pathology specimens has undergone marked expansion in the past decade. The field of LVO clots, in particular, is increasingly moving beyond conventional histology and toward molecular and next-generation techniques, which enable more detailed and comprehensive tissue characterization [88]. These advancements, particularly in individual -omic and multiomic approaches, offer substantial potential to revolutionize our understanding of clot composition and underlying stroke etiology [88].

Although promising, literature on -omic and multiomic approaches remains limited, with most studies including only small patient cohorts. To ensure a comprehensive review of the available data, we searched the MEDLINE and EMBASE databases (query date: 23 August 2025) using the MeSH/Emtree terms “Ischemic stroke” combined with “Thrombectomy” (or “Mechanical Thrombectomy for Emtree) and [“Metabolomics” or “Proteomics” or “Gene Expression Profiling” (MeSH term for transcriptomic)]. Titles and abstracts were screened for relevance to stroke etiology, and the results were cross-referenced with the most recent review on the subject [88] to ensure completeness.

### 5.1. Proteomics

Proteomic analysis utilizes global and targeted mass spectrometry to quantify the levels of proteins in a sample. Individual protein levels can be then compared, or bioinformatic analyses can be applied to identify patterns of protein activation or suppression [89]. Proteomics has been utilized to detect the proteins and protein–protein interactions involved in the pathogenesis of stroke clots [90], as well as associations of different proteins to a variety of clinical features including fibrinolytic activity [91].

Several studies have employed proteomic techniques to attempt to find connections between protein level patterns and stroke etiology. Darganzanli et al. applied mass spectrometry and machine learning to identify proteomic distinguishing LAA from CE clots [92]. They found that coagulation factor XIII, which catalyzes the last step of the coagulation cascade, was most consistently different between the two etiologies. When combined with a history of cardiac failure, a trio of proteins (coagulation factor XIII, eukaryotic translation initiation factor 2 subunit 3, and Ras GTPase-activating-like protein IQGAP2) yielded a classification accuracy of 97% [92]. Rossi et al. conducted a similar analysis and identified 14 proteins with significantly different concentrations between the two etiologies [93]. Four proteins involved in the ubiquitin–proteasome pathway, coagulation, or plasminogen activation were enriched in LAA clots, whereas ten proteins involved in the ubiquitin–proteasome pathway, cytoskeletal remodeling of platelets, platelet adhesion, and coagulation were more abundant in CE clots. In a recent proteomic analysis, Kim et al. found a number of proteins and pathways that were specifically upregulated in LAA clots, CE clots, cancer-related clots, and cryptogenic clots [94]. Similarly, they found elevated levels of proteins involved in the thrombosis and hemostasis pathway (PLEK, ROCK2, TLN1, and RAB14) in CE clots, whereas proteins involved in the ubiquitin–proteasome pathway and atherosclerosis progression (e.g., CD59, LAMP1, and ELANE) were found in abundance in LAA clots. Other studies examining the relationship between proteomics and stroke etiology are detailed in Table 4. A major limitation across these investigations lies in the difficulty of translating proteomic analyses to laboratory tools available in the clinical setting. Moreover, the small size (or absence) of an external validation cohort currently restricts their clinical applicability.

### 5.2. Metabolomics

Rooted in systems biology, metabolomics is the study of small-molecule metabolites and lipids, which is performed after larger proteins are separated from the samples. While peripheral metabolomic profiling in stroke has been widely studied, LVO-specific metabolomic analysis remains in its early stages, with only few publications to date [88,95]. Using liquid chromatography–mass spectrometry, Martha et al. analyzed five clots and identified the top ten most frequent metabolites as various glycerophospholipids and fatty acids [96]. Li et al. utilized quadrupole time-of-flight mass spectrometry and identified six metabolites that, when combined with a machine learning random forest classifier, yielded a predictive model with an area under the curve (AUC) of 0.89 [97]. The metabolites enriched in LAA clots were diglyceride (DG) (18:3/24:0), DG (22:0/24:0), phytosphingosine, and galabiosylceramide (18:1/24:1); while those enriched in CE clots were triglyceride (15:0/16:1/o-18:0) and glucosylceramide (18:1/24:0). Using H&E and IHC, Osakada et al. identified a localized pattern with 4-hydroxyl-2-nonenal, an oxidative stress marker, in LAA clots [98]. Suissa et al. combined proteomics and metabolomics to analyze 59 clots [99]. Their proteomic analysis revealed elevated levels of fibrinogens α, β, γ—components of fibrin—in CE clots, consistent with prior histologic findings. Interestingly, glycophorin-A, a marker for RBCs, was also significantly more abundant in CE clots. Using an integrated proteomic and metabolomic approach, the authors developed a predictive model with 100% sensitivity and 85.7% specificity in distinguishing CE from LAA clots. Furthermore, in cases initially classified as cryptogenic, the model predicted a CE source in all patients who were later diagnosed with atrial fibrillation at their 3-month follow-up.

Table 4 summarizes the available literature pertaining to LVO etiologies and proteomic and/or metabolomic analyses.

**Table 4:** Literature on stroke etiologies and clot proteomics/metabolomics.

Reference	Sample Size	Technique	Findings
Darganzanli, 2020 [92]	60 (32 CE, 28 LAA)	Proteomic with Nano- LC-MS. Dataset then analyzed using support vector machine (SVM) learning method.	Protein trios allowing 88% accuracy of correct classification are coagulation factor XIII + eukaryotic translation initiation factor 2 subunit 3 & Ras GTP-ase-activating-like protein IQGAP2. F-actin-capping protein & myosin light chain kinase. Septin-7 & gamma-adducin. When integrating protein trio 1 + history of cardiac failure + protein concentration, the accuracy improved to 97%.
			*Specific limitations:* Proof-of-concept study. Does not include external validation.
Suissa, 2021 [99]	41 (34 CE, 7 LAA)	Multiomic (combined proteomic and metabolomic approach)	Using the combined proteomic and metabolomic signature, the authors’ model achieved 100% sensitivity and 85.7% specificity of predicting CE source. External validation performed on patients initially classified as cryptogenic achieved 100% prediction in the rate of new atrial fibrillation diagnosis at 3 months.
			*Specific limitations:* Small validation cohort (7 patients).
Abbasi, 2021 [100]	48 (25 CE, 23 LAA)	Proteomic with RPPA	CE clots have more diverse and abundant protein linkages between PPAR-gamma and arginase-1, CD63, CD234, PKCαβ Thr 638/641, and vWF.
			*Specific limitations*: Descriptive observational findings only. Does not include predictive modeling.
Lopez-Pedrera, 2023 [101]	18 (9 CE, 9 LAA)	Proteomic with nano-LC-MS	26 proteins were differentially abundant between CE and LAA clots: - CE: predominance of Protein S100-P, vitronectin, ceruloplasmin, clusterin, histone cluster 2 H3 family member a, 26S proteasome regulatory subunit 10B, antithrombin-III, 26S proteasome regulatory subunit 4, glutamate-cysteine ligase regulatory subunit, elongation factor Tu, mitochondrial, alpha-1-antichymotrypsin, U6 snRNA-associated Sm-like protein LSm2, apolipoprotein A-I, C-1-tetrahydrofolate synthase, cytoplasmic, 6-phosphogluconolactonase, elongation factor 1-alpha 1, small ribosomal subunit protein uS3, S-formylglutathione hydrolase, adenine phosphoribosyltransferase, nucleosome assembly protein 1-like 1. - LAA: predominance of ubiquitin-like-conjugating enzyme ATG3, blood group Rh(D) polypeptide, fibrinogen alpha chain, solute carrier family 2, facilitated glucose transporter member 1, keratin type II cytoskeletal 1, chloride intracellular channel protein 4.
			*Specific limitations:* Descriptive observational findings only. Does not include predictive modeling or external validation.
Li, 2023 [97]	48 (26 CE, 22 LAA)	Metabolomic with UPLC-QTOF-MS	6 metabolites were differentially abundant in CE and LAA clots and selected by machine learning model: - CE: TG (15:0/16:1/o-18:0) and glucosylceramide (18:1/24:0). - LAA: DG (18:3/24:0), DG (22:0/24:0), phytosphingosine, and galabiosylceramide (18:1/24:1). Random forest model achieved AUC of 0.89 for discriminating between CE and LAA origins in the external validation cohort.
			*Specific limitations:* Validation cohort does not include cryptogenic patients, only those with LAA and CE clots.
Rossi, 2022 [93]	31 (16 CE, 15 LAA)	Proteomic with LC-MS/MS	14 proteins were differentially abundant between CE and LAA clots: - CE: myosin-9, coronin-1C, actin-related protein 2/3 complex subunit 2, platelet glycoprotein Ib alpha chain, platelet glycoprotein IX, protein disulfide-isomerase A6, Valosin-containing protein (VCP), ubiquitin-like modifier-activating enzyme 1, coagulation factor XIII A chain, Ras-related protein RAB-27B - LAA: ubiquitin-60S ribosomal protein L40, ubiquitin-conjugating enzyme E2, ubiquitin-conjugating enzyme E2 variant 1, and fibrinogen alpha chain.
			*Specific limitations:* Descriptive observational findings only. Does not include predictive modeling or external validation.
Kim, 2025 [94]	27 (17 CE, 6 LAA, 4 CR)	Proteomic with LC-MS/MS	- Proteins upregulated in CE clots are involved in actin cytoskeleton organization, supramolecular fiber organization, platelet aggregation, hemostasis, coagulation, apoptotic processes, and the tricarboxylic cycle. - Proteins upregulated in LAA clots are involved in ubiquitination, ubiquitin-proteasome system, endothelial migration, and atheroma formation. Using machine learning, the predictive model has a PPV of 75% in predicting etiology in the cryptogenic/undetermined group during the follow-up period.
			*Specific limitations*: Lower PPV, with a small validation cohort (8 patients).

CE, cardioembolic; CR, cancer-related; DG, diglyceride; LAA, large-artery atherosclerosis; LC, liquid chromatography; MS, mass spectrometry; PPV, positive predictive value; RPPA, reverse-phase protein array; TG, triglyceride; UPLC-QTOF-MS, ultra-performance liquid chromatography coupled with quadrupole time-of-flight mass spectrometry; vWF, von Willebrand factor.

### 5.3. Transcriptomics

Transcriptomic analysis uses next-generation sequencing to quantify mRNA levels and measure gene expression profiles. This approach has been greatly advanced by exploration into genetic determinants of AIS. In particular, genome-wide association studies (GWAS) have demonstrated correlation of specific genetic loci with stroke etiology, risk factors, and long-term functional outcome [102,103,104]. These studies have identified key transcriptional pathways implicated in atrial fibrillation, inflammation, lipid metabolism, and vascular remodeling, all with significant associations with AIS [104]. For instance, two genes involved in cardiac development, PITX2 and ZFHX3, have been consistently associated with atrial fibrillation and an increased incidence of CE strokes [103,104]. Such genetic insights can, in turn, be leveraged to guide transcriptomic analyses of retrieved clots to enable interrogation of specific pathways linked to the genes of interest [105]. Transcriptomic expression profiles can be defined for a sample as a whole (bulk RNA-seq), segregated into single cells (scRNA-seq), or spatially within a sample (spRNA-seq). The ”big data” generated from such methods is then analyzed according to known gene expression clusters or pathways to dissect the patterns of activation or inhibition in specific disease contexts [106].

In an early effort to use mRNA profiling to differentiate stroke etiology, Baek et al. used quantitative real-time polymerase chain reactions on 82 MT clots, focusing on gene expression markers for inflammation [105]. They found that IL-1β, a cytokine whose release can be triggered by local inflammasomes, was upregulated in the nine LAA cases. In a more recent analysis, Tutino et al. used bulk RNA-seq to analyze 38 clots [107]. They found that LAA clots tended to have stronger expressions of genes involved in oxidoreduction and T cell signaling, whereas CE clots had much denser expression of immunological pathways involving neutrophils and myeloid leukocytes. This trend in gene ontology, where LAA clots tend to feature more adaptive immunity activation and CE clots more innate immunity activation, closely aligns with the WBC subcomponent analyses previously discussed [43,73]. Interestingly, cryptogenic clots did not necessarily cluster towards either the LAA or CE expression, possibly suggesting heterogenous etiology [107].

In a recent work from our group, LAA clots were found to be enriched in B cells, CD4+ T cells, dendritic cells, and macrophages [108]. Conversely, CE clots had more CD8+ T cells, monocytes, NK cells, and neutrophils. Using scRNA-seq, we found that CE clots had upregulation of genes involved in signaling pathways related to immunogenic cell death, Th1, RHOA, actin nucleation by ARP-WASP complex, ILK, NET, and hepatic fibrosis, in all cell types. On the other hand, macrophages from LAA clots showed upregulation of the Th1 pathway, multiple sclerosis signaling pathway, and phagosome formation, while CD8+ T cells from CE clots were enriched in PD-1, PD-L1 cancer immunotherapy, and CTLA4 signaling. Beyond predicting stroke etiology, Santo et al. identified 25 differentially expressed genes from the transcriptome data to be significant predictors of 90-day mRS [109]. In a subsequent study, the same group achieved 94% accuracy in predicting good long-term outcomes using a hybrid model integrating histomic. and transcriptomic clot features [110]. Table 5 summarizes the available literature pertaining to LVO etiologies and transcriptomic analyses.

Compared to conventional histology, -omic and multiomic approaches promise superior sensitivity and specificity in distinguishing stroke etiologies, particularly when integrated with machine learning and clinical data. However, this field remains in its early stages, and external validation in larger cohorts is required to clarify the clinical utility of these approaches. From a practical standpoint, the high cost, extended processing time, and limited availability (primarily restricted to select academic centers), pose significant barriers to widespread clinical adoption. Continued advancement in technology, protocol standardization, and access will be essential in translating these promising approaches into routine clinical practice.

## 6. Summary of Current Evidence

**Figure 2:**
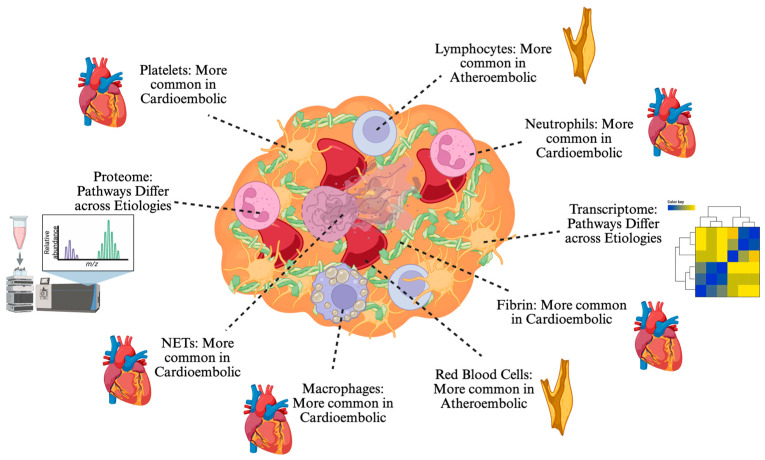
A simplified schematic summarizing the published relationships between clot composition and stroke etiology to date. Where indicated, different components have been found to be more abundant in clots stemming from atheroembolic or cardioembolic etiologies, although significant heterogeneity exists. Proteomic and transcriptomic studies have shown that the associated pathways are distinct between stroke etiologies, although further work is needed to consistently define and understand these differences, their implications, and clinical utility. Created in BioRender. Sujijantarat, N. (2025) https://BioRender.com/rjmuicv (accessed on 9 August 2025).

A review of the current literature allows several observations regarding the relationship between clot composition and stroke etiology (Figure 2, Table 6). Clots from LAA tend to have greater RBCs on histological analysis than those of CE origin [39]. Conversely, CE clots tend to have a greater proportion of fibrin and platelets [39]. Findings on total WBC content between the two etiologies are conflicting, with any reported statistically significant differences too small to be clinically meaningful [39]. When WBC subpopulations are considered, CE clots tend to have greater representation of innate immunity such as neutrophils [64] and monocytes [44], whereas LAA clots have a greater lymphocytic filtration [74]. Similarly, CE clots have more NETs [74] and extracellular DNA [40]. In terms of gene expression, CE clots demonstrate upregulation of genes involved in inflammation and neutrophil activity [107], while LAA clots are characterized by T cell and oxidative signaling [107]. Although results are promising in metabolomics and proteomics, several studies to date have yet to produce common pathways that definitively differentiate clot etiologies [88,92,94].

Several studies using conventional histology have shown that cryptogenic clots share features more consistent with CE clots [20], including a greater quantity of platelets and fibrin and lower RBCs [40]. This has led to the speculation in several studies that cryptogenic strokes may reflect undiagnosed cardioembolic events [20,40,45], including atrial fibrillation not discovered during the hospital stay [19]. However, these trends are not universal across studies and have not been clearly supported by analyses of WBC subcomponents [43] or transcriptomic [107] or proteomic profiles [88].

## 7. Limitations and Future Directions

Although clot composition analysis offers a promising avenue for investigating stroke etiology, several challenges currently limit its routine clinical application. First, the pro-thrombotic large-artery and cardiac environments may lead to the formation of clots that appear morphologically, histologically, and even molecularly similar [111]. Second, clots formed by a similar etiology can demonstrate significant variability in all of these categories [39]. Third, spatial heterogeneity within the clots, whether intrinsic or secondary to endovascular intervention, may confound interpretation of clot composition [112]. Finally, inherent methodological limitations constrain the utility of clot composition analysis. Several studies suggest that RBC-rich clots are more susceptible to fibrinolysis, likely due to improved drug penetration within the loosely organized RBC clusters compared to platelet/fibrin-rich clots [49,113,114,115,116]. Such clots may lyse before they can be retrieved via MT. On the other hand, stroke due to small-vessel disease and highly recalcitrant clots that withstand both fibrinolysis and MT also remain unavailable for analysis. Because clot composition studies are inherently restricted to specimens retrievable via MT, our current understanding is shaped by only a subset of clots resistant to fibrinolysis yet amenable to mechanical extraction. This selection bias may result in overrepresentation of certain structural and molecular features and underrepresentation of others. Moreover, fibrinolysis itself may alter the composition of successfully retrieved clots, further confounding the interpretation [20].

To date, multiomic data have shown the greatest promise in predicting stroke etiology. Machine learning offers a powerful means of handling the computational demands of pattern recognition and trend detection inherent to these large, complex datasets. In the future, such approaches could integrate the full spectrum of data from a given clot (histologic, proteomic, metabolomic, and/or transcriptomic) and apply established LAA and CE signatures to aid in classification. Importantly, these models could be designed to account for inherent heterogeneity within the stroke subtypes and improve diagnostic specificity. Early studies have already demonstrated high accuracy in differentiating LAA from CE clots and in reclassifying some cryptogenic strokes as CE. Although barriers such as cost, processing time, and limited availability currently preclude bedside implementation, continued advances in these areas may allow integrated profiles to become a clinically deployable tool for routine clinical decision-making.

Beyond determining stroke etiology, clot composition analysis is increasingly explored for a range of clinical applications. Multiple studies have examined associations between clot histology and both acute recanalization success [37,69] and long-term outcomes [43,58,109,110]. Others have investigated correlations between radiologic clot characteristics and histologic and molecular features [117,118,119]. For example, Hyperdense Artery Sign (HAS) on non-contrasted CT has demonstrated correlation with RBC-rich phenotype and improved recanalization rates [117]. On MRI, Susceptibility Vessel Sign (SVS), representing hypointensity at the site of the clot, has been shown to be associated with lower platelet content and improved functional outcome [118]. However, a recent large study of 1430 patients found no significant association between clot composition and MT efficacy [120]. In contrast, novel imaging techniques such as oxygen extraction fraction mapping and quantitative susceptibility mapping do not focus on the clot itself but on tissue-level changes that may relate to stroke etiology and its recovery potential [121]. Together, these analyses advance our understanding of the molecular and cellular mechanisms underlying stroke pathophysiology [30,90], with potential implications for biomarker discovery, secondary prevention, and the development of targeted therapeutics [88,89,90,111,122].

## 8. Conclusions

Clot composition analysis, particularly through the use of next-generation -omic and multiomic approaches, offers a novel opportunity to examine the underlying pathology and etiologic “story” of each clot. By providing molecular and structural insights, these approaches can illuminate the mechanisms of clot formation and inform more precise strategies for secondary stroke prevention. Growing evidence of distinct compositional differences between LAA and CE clots strengthens our understanding of these entities and underscores the diagnostic value of clot profiling. In cryptogenic stroke, where conventional workup often falls short, clot analysis holds promise as an adjunctive tool to identify occult etiologies and guide targeted interventions, including anticoagulation. Taken together, these findings position clot composition analysis as an emerging bridge between specimen analysis and actionable, personalized stroke care.

## Figures and Tables

**Table 1:** Trial of Org 10,172 in Acute Stroke Treatment (TOAST) classification of subtypes of acute ischemic stroke [17].

1	Large-artery atherosclerosis (embolus/thrombosis)
2	Cardioembolism (high-risk/medium-risk)
3	Small-vessel occlusion (lacune)
4	Stroke of other determined etiology
5	Stroke of undetermined etiology (“cryptogenic stroke”)
	a. Two or more causes identified
	b. Negative evaluation
	c. Incomplete evaluation

**Table 2:** Common staining techniques for detection of macrocellular components and white blood cell subpopulations of the clot.

Component	Staining Technique
Red blood cells	H&E staining [48], MSB (selective quantification) [49], IHC CD235a antigen [50,51]
Fibrin	H&E staining [48], MSB (selective quantification) [49], Picro-Mallory (can also detect fibrin maturity in thrombi) [21], Ladewig trichrome [52]
Platelets	MSB [49], CD41 [53], CD42b [38,54,55,56], CD61 [57]
White blood cells	H&E staining [48], IHC CD45 antigen [58,59], neutrophil elastase [58], neutrophil myeloperoxidase [51]
Monocytes	Ly6G [60], CD14 [58], CD15 [61], CD68 [44,62,63]
Granulocytes	Ly6G [60]
Neutrophils	Ly6G [60], CD15 [61], CD66b [47,64] NET-associated: citrullinated histones, caspase-1, and apoptosis-associated speck-like protein [65], granular neutrophil proteins (MPO), extracellular DNA [47]
Eosinophil	CD15 [61]
T-lymphocyte	CD3 [66], CD4 [63]
B-lymphocyte	CD20 [44]
Coagulation proteins	antibodies against vWF [50,56,61,63,67]
tPA	plasminogen activator inhibitor-1 [50], protease nexin-1 [50]
Elastic collagen fibers	Elastica van Gieson [52,68]
Hemosiderin/iron	Prussian blue [52]
Calcifications	Von Kossa [52,68]
Collagen	Masson’s trichrome [21]

H&E, hematoxylin and eosin; IHC, immunohistochemical staining; MPO, myeloperoxidase; MSB, Martius Scarlet Blue; NET, neutrophil extracellular trap; tPA, tissue plasminogen activator; vWF, von Willebrand factor.

**Table 3:** Summary of major WBC subcomposition differences comparing LAA and CE clots.

WBC Type	LAA Clot	CE Clot	References
**Innate Immune Cells**	**↓ (less enriched)**	**↑ (more enriched)**	[44,52,58,64,74]
CD68+ (Macrophages) 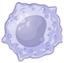	↓	↑	[44,52]
NE+ MPO+ Neutrophils 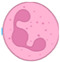	↓	↑	[58,64,74]
**Adaptive Immune Cells**	**↑**	**↓**	[66,72,73,74]
CD3+ (lymphocytes) 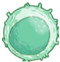	↑	↓	[66,74]
CD4+ (Helper T cells) 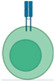	↑	↓	[72,73]

CE, cardioembolism; LAA, large-artery atherosclerosis; MPO, myeloperoxidase; NE, neutrophil elastase; WBC, white blood cell.

**Table 5:** Literature on stroke etiologies and clot transcriptomics.

Reference	Sample Size	Technique	Findings
Tutino, 2023 [107]	38 (21 CE, 6 LAA, 5 other determined cause, 6 cryptogenic)	Paired-end RNA-seq	- Compared to LAA, genes upregulated in CE clots include those involved in immune responses and cellular localization. - Compared to CE, genes upregulated in LAA clots include those involved in reactive oxygen species and oxidase activity. Cryptogenic cases do not cluster towards only one group.
			*Specific limitations:* Descriptive observational findings only. Does not include predictive modeling or external validation. Validation cohort contained low RNA concentrations, and only 3 DEGs could be tested.
Renedo, 2025 [108]	12 (6 CE, 4 LAA, 2 venous)	scRNA-seq	- Genes upregulated in CE clots: GZMH, GZMB, S100A4, FCGBP2, HLA-A, TIMP1, CLIC1, and IFITM2. - Genes upregulated in LAA clots: CD74, HLA-DRB1*01, HTRA1, C1Q, CD81, and CR1.
			*Specific limitations:* Small sample size. Descriptive observational findings only. Does not include predictive modeling or external validation.

CE, cardioembolic; DEG, differentially expressed genes, LAA, large-artery atherosclerosis; scRNA-seq, single-cell RNA sequencing.

**Table 6:** Summary of current evidence linking various clot subcomponents to stroke etiology.

Clot Components	LAA Clot	CE Clot
RBCs	↑ (more enriched)	↓ (less enriched)
Platelets/Fibrin	↓	↑
Innate Immune Cells	↓	↑
Adaptive Immune Cells	↑	↓
Multiomic pathways	Multiple unique associations	Multiple unique associations

CE, cardioembolism; LAA, large-artery atherosclerosis; RBCs, red blood cells.
